# A modular computational framework for automated peak extraction from ion mobility spectra

**DOI:** 10.1186/1471-2105-15-25

**Published:** 2014-01-22

**Authors:** Marianna D’Addario, Dominik Kopczynski, Jörg Ingo Baumbach, Sven Rahmann

**Affiliations:** 1 Collaborative Research Center SFB 876, TU Dortmund University, Dortmund, Germany; 2 B & S Analytik, BioMedical Center, Dortmund, Germany; 3 Genome Informatics, Institute of Human Genetics, Faculty of Medicine, University of Duisburg-Essen, Essen, Germany

**Keywords:** Ion mobility spectrometry, Peak detection, Signal processing, Automated pipeline

## Abstract

**Background:**

An ion mobility (IM) spectrometer coupled with a multi-capillary column (MCC) measures volatile organic compounds (VOCs) in the air or in exhaled breath. This technique is utilized in several biotechnological and medical applications. Each peak in an MCC/IM measurement represents a certain compound, which may be known or unknown. For clustering and classification of measurements, the raw data matrix must be reduced to a set of peaks. Each peak is described by its coordinates (retention time in the MCC and reduced inverse ion mobility) and shape (signal intensity, further shape parameters). This fundamental step is referred to as *peak extraction*. It is the basis for identifying discriminating peaks, and hence putative biomarkers, between two classes of measurements, such as a healthy control group and a group of patients with a confirmed disease. Current state-of-the-art peak extraction methods require human interaction, such as hand-picking approximate peak locations, assisted by a visualization of the data matrix. In a high-throughput context, however, it is preferable to have robust methods for fully automated peak extraction.

**Results:**

We introduce PEAX, a modular framework for automated peak extraction. The framework consists of several steps in a pipeline architecture. Each step performs a specific sub-task and can be instantiated by different methods implemented as modules. We provide open-source software for the framework and several modules for each step. Additionally, an interface that allows easy extension by a new module is provided. Combining the modules in all reasonable ways leads to a large number of peak extraction methods. We evaluate all combinations using intrinsic error measures and by comparing the resulting peak sets with an expert-picked one.

**Conclusions:**

Our software PEAX is able to automatically extract peaks from MCC/IM measurements within a few seconds. The automatically obtained results keep up with the results provided by current state-of-the-art peak extraction methods. This opens a high-throughput context for the MCC/IM application field. Our software is available at http://www.rahmannlab.de/research/ims.

## Background

While ion mobility (IM) spectrometry (IMS) is an established technology to detect volatile organic compounds (VOCs) in the air or exhaled breath, the more recent combination with multi-capillary columns (MCCs) has opened new applications in biotechnology and medicine, consider Koczulla *et al.*[1] and Armenta *et al.*[2]. The analytes, metabolites present within exhaled breath, are pre-separated using the MCC, analogously to gas chromatography (GC) before mass spectrometry (MS).

VOCs from the human metabolism in exhaled breath may hint at certain diseases. Applications to diagnosis of lung cancer, chronic obstructive pulmonary disease (COPD) or sarcoidosis have already been reported [3-7].

### MCC/IM measurements and peaks

A single measurement with an IM spectrometer takes about 100 ms, using nitrogen or synthetic air as drift gas. For a sample pre-separated by an MCC, an IM spectrum is captured periodically at several different retention times, e.g. each 100 ms for up to 10 minutes. The retention time *r* is the time a compound needs to pass the MCC.

**Figure 1:**
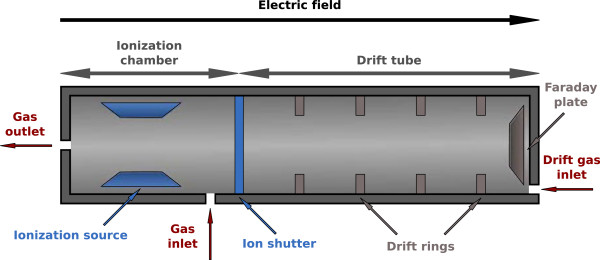
**Schematic cross section of an IM spectrometer.** Analyte compounds pre-separated by the MCC are ionized in the ionization chamber. The ions are accelerated by an electric field and move through the drift tube. They cause a voltage change when colliding with the Faraday plate; this is the measured signal.

The drift time *t*^′^ is the time a compound needs to drift through the IM spectrometer and is influenced by parameters such as drift tube length, intensity of electric field as well as temperature or ambient pressure. Figure 1 illustrates a It is thus advantageous to consider a normalized quantity: the reduced inverse mobility *t* in Vs/cm^2^. Each IM spectrum (at a specific MCC retention time *r*) provides a signal intensity (ion count, measured as voltage change on a Faraday plate; for technical details see [8]) for each value of *t*.

We obtain a two-dimensional IM spectrum-chromatogram (IMSC) S:R×T→ℤ with retention times *r*∈*R*, inverse mobilities *t*∈*T*, and signal intensities *S*(*r*,*t*)∈*ℤ* (measured as unsigned 12-bit values). In practice, we have equidistant points on both retention time and inverse mobility axes; therefore we may assume that *R*={1,…,*m*} and *T*={1,…,*n*}, where these index values correspond to actual times and reduced inverse mobilities.

**Figure 2:**
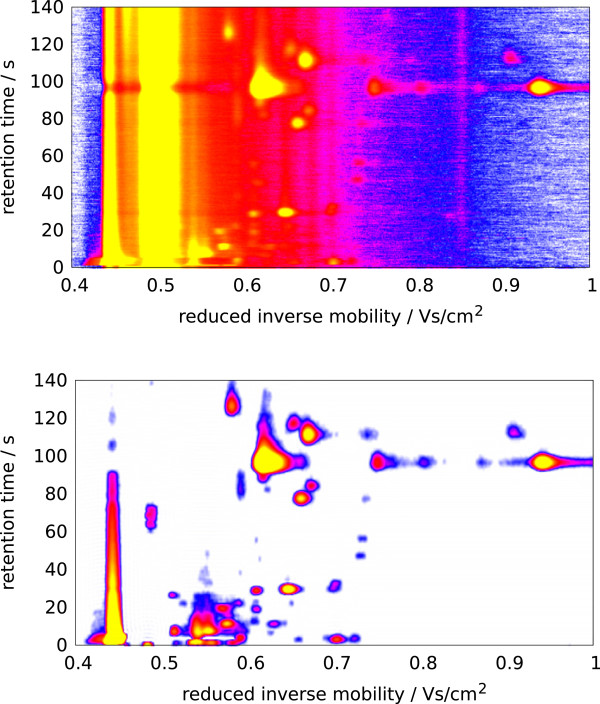
**IMSC visualized before and after preprocessing.Top:** Heat map of a raw IMSC. X-axis: reduced inverse mobility *t* in Vs/cm^2^; Y-axis: retention time *r* in seconds; signal: white (lowest) < blue < purple < red < yellow (highest), reactant ion peak (RIP) at *t* = 0.48Vs/cm^2^. **Bottom:** IMSC after preprocessing using bc-dn-s (see text).

We call a single row or spectrum *S*_*r*_ at a retention time *r* an *IM spectrum*. A single column *S*_·,*t*_ at a certain *t* is called *IM chromatogram*. The whole matrix *S* is the IMSC. Regions of *S* with a high signal intensity are called *peaks*. An IMSC can be visualized as a heat map (Figure 2). In every IMSC, the *reactant ion peak* (RIP) produced by the ionisation of the drift gas is visible as a high-intensity chromatogram at a reduced inverse mobility of approximately *t*=0.48 Vs/cm^2^. When additional analyte ions occur, the RIP is reduced and may even disappear if the analyte concentrations are extremely high. We describe each peak with a set of (at least three) descriptors, which are its coordinates (*r*,*t*) and signal intensity *s* (for example *s*=*S*_*r*,*t*_). Additional parameters may describe the peak shape or alternative signal values. For typical analysis algorithms, a triple (*r*,*t*,*s*) suffices, where we use *s* as shorthand for any signal at or around (*r*,*t*), how ever computed.

The position and intensity of peaks indicates the presence and concentration of certain VOCs. Peaks behaving differentially (presence vs. absence or quantitative difference) in two classes of measurements (i.e., patients vs. controls) may represent potential biomarkers that can hint at specific diseases.

### The need for automated peak extraction: our contributions

The fundamental step of peak extraction from a raw IMSC is the basis for all subsequent data mining classification steps [9]. Given a set of measurements, a domain expert assisted by visualization software (such as VisualNow from B&S Analytik, Dortmund, Germany) is able to interactively pinpoint peak locations within a few minutes. An experienced expert can often distinguish weak signals from noise.

MCC/IMS technology has matured to a point where it is applied to automated monitoring [10] and moves towards a high-throughput domain. Here, interactive expert-driven and computer-assisted peak extraction is no longer possible. To a lesser extent, the same situation holds true in exploratory medical studies, where the amount of available measurements increases beyond human analysis capabilities. Therefore, automated peak extraction methods are urgently required. As another advantage, they offer better reproducibility and increased speed. However, they may make certain assumptions about the data and lack in adaptability.

We here provide a modular automated peak extraction framework. The task of peak extraction is divided into four steps that are performed in sequence. Each step allows us to use different specific methods, implemented as separate modules. Each (reasonable) combination of modules, together with individual module parameters, specifies a concrete peak extraction pipeline and transforms an IMSC into a list of peaks.

In Section ‘Methods’ we introduce the peak extraction framework, enumerating briefly all pipeline steps. The next three sections (Sections ‘Modules for preprocessing, Modules for peak candidate detection, Modules for peak picking, A module for peak modeling (pme)’) explain the available modules for each step in detail. Information on the software architecture and implementation is given in Section ‘Architecture and implementation’. Results on a complete dataset are provided in Section ‘Results’, where we compare the different combinations with each other and to manual peak extraction. Section ‘Discussion and conclusion’ concludes.

## Methods

### Framework overview

**Figure 3:**
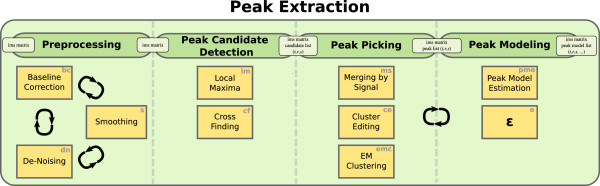
Steps and modules of the peak extraction framework PEAX.

We present a framework for automatic processing of an MCC/IM measurement (ion mobility spectrum-chromatogram, IMSC) to discover and quantify all present peaks. The peak extraction process is divided into four steps (Figure 3). Each step can be implemented by different modules represented by the yellow boxes containing an abbreviation for each module name. Each resulting pipeline requires a single IMSC as input and outputs a list of peaks. Each peak is represented at least by the following information: name of the measurement, an automatically given peak ID, reduced inverse mobility, retention time, signal value, reduced inverse mobility index and retention time index. Knowing the name of measurement for each peak is convenient when comparing several peak lists from different measurements. We now discuss the four distinct steps.

*Preprocessing* transforms a (raw) IMSC into another (processed) IMSC, i.e., no data reduction or peak extraction takes place. Raw IMSCs are noisy and include the confounding RIP. To remove both noise and the RIP, we describe three modules: *Baseline Correction* (bc), *De-Noising* (dn) and *Smoothing* (s); every module’s input and output is an IMSC. Baseline Correction (bc) handles the RIP (and the baseline in general), removes it, and uncovers underlying peaks. De-Noising (dn) estimates the probability of a data point belonging to noise in order to remove the noise. Smoothing (s) applies a smoothing filter. The order of execution is commutable, but none of these modules can be omitted. Figure 2 shows a measurement before and after preprocessing.

*Peak candidate detection* finds a list of potential peaks within the preprocessed IMSC. We implemented two alternative modules called *Local Maxima* (lm) and *Cross Finding* (cf). The input of either module is a processed IMSC, and the output is a list of candidate peaks, which is further refined in the next step. Local Maxima finds local maxima within the two-dimensional IMSC, while Cross Finding searches for zeros in the first partial derivatives with respect to both retention time and reduced inverse mobility.

*Peak picking* examines the proposed candidates and generates the final list of extracted peaks. We have so far implemented three modules. All three methods calculate a representative peak for a set of peaks whose positions are too close to be considered distinct. *Merging by signal intensity* (ms) is a basic method considering the distance between two candidates; it picks highest signal peak from a set of close peaks within a surrounding box of given size. *Cluster editing* (ce) discovers peak clusters by solving the cluster editing problem and returns the peak with highest intensity of each cluster as representative. *EM Clustering* (emc) works similarly, but discovers peak clusters using the EM algorithm.

*Peak modeling* is an optional final step that can be used to estimate additional peak parameters, describing the shape and position more precisely. A module called *Peak model estimation* (pme) has been implemented. In time-critical applications this step is generally omitted by using the “empty module” *ε*.

The Peak Modeling step can be exchanged with the Peak Picking step, meaning that each candidate peak is modeled, and picking is done on the modeled candidate peaks. In the following section we discuss all modules in detail and introduce several parameters, remark that user adjustable parameters are emphasized.

### Mixture models and expectation maximization

Several of our modules use mixture modeling, i.e., the data is viewed as a realization of a mixture distribution

$$f(x\;|\;\theta )=\overset{C}{\underset{c=1}{\sum }}\;\omega_{c}\;f_{c}(x\;|\;\theta_{c}),$$

where *c* indexes the *C* different components, *θ*_*c*_ denotes all parameters of distribution *f*_*c*_, and *θ*=(*θ*_1_,…,*θ*_*c*_) is the collection of all parameters. We allow that the distributions *f*_*c*_ are of different types, e.g., a uniform and a Gaussian one. The mixture coefficients *ω*_*c*_ satisfy *ω*_*c*_≥0 for all *c*, and $\underset{c}{\sum }\;\omega_{c}=1$.

The goal of mixture model analysis is to estimate the mixture coefficients *ω*=(*ω*_*c*_) and the individual model parameters *θ*_*c*_ (whose number and interpretation depends on the parametric distribution *f*_*c*_). Since this maximum likelihood problem is non-convex, iterative locally optimizing methods such as the Expectation Maximization (EM) algorithm [11] are frequently used. The EM algorithm consists of two repeated steps: The E-step (expectation) estimates the expected membership of each data point *x* in each component and then *ω*, given the current model parameters *θ*. The M-step (maximization) estimates maximum likelihood parameters *θ*_*c*_ for each parametric component *f*_*c*_ individually, using the expected memberships as hidden variables that decouple the model. As the EM algorithm converges towards a local optimum of the likelihood function, it is crucial to choose reasonable starting parameters for *θ*. For details, we refer to our previous work on peak modeling (contained herein as the module Peak Model Estimation (pme)), where we describe how to apply the EM algorithm to a mixture of Inverse Gaussian distributions to infer peak shape parameters [12].

## Modules for preprocessing

### Baseline correction (bc)

**Figure 4:**
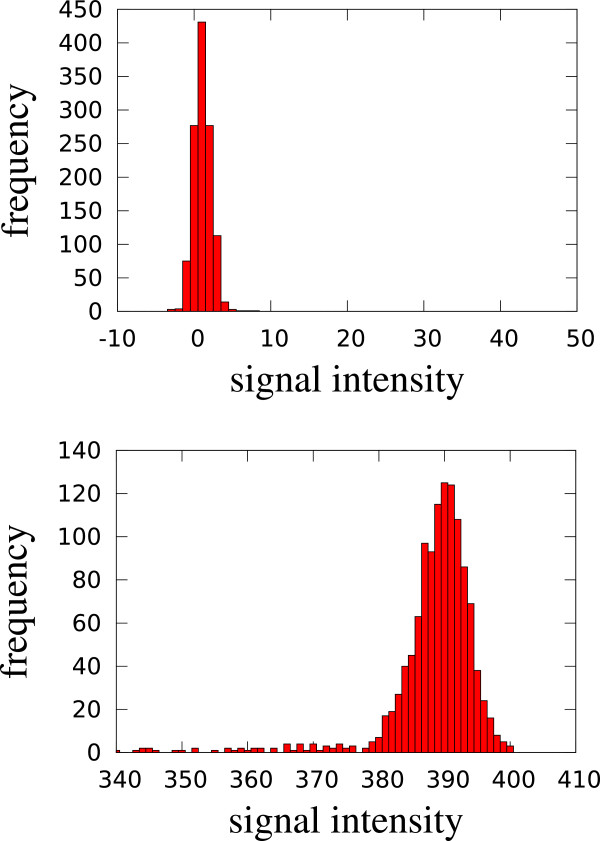
**Histograms (y-axis: frequency) of signal intensities (x-axis) of two chromatograms, a typical one (top) and a RIP chromatogram (bottom).** The prominent intensity peak is modeled by a Gaussian distribution.

Intuitively and informally, a baseline spectrum *B*=(*B*_*t*_)_*t*∈*T*_ is defined such that *B*_*t*_ is a typical or insignificantly high value at reduced inverse mobility *t* when considering the whole measurement. Formally, for each reduced inverse mobility *t*, we consider a histogram *H*_*t*_ with bin size 1 of the chromatogram *S*_·,*t*_, i.e., *H*_*t*,*i*_ is the number of data points with intensity *i* in the chromatogram. Bader [13] presented a method that assumes a log-normal model as baseline and estimate its parameters before subtracting from spectrum. We developed a new method since Baders method does not erase the whole RIP. In a typical chromatogram (mostly noise, one or a few peak(s), no RIP), most intensities are at noise level, so the most prominent peak in the histogram indicates that level. In a RIP chromatogram, the most prominent peak corresponds to the RIP level (Figure 4). In both cases and in the intermediate ones as well, we model the most prominent peak of the histogram by a Gaussian distribution and the remainder by the uniform distribution between lowest and highest observed intensity.

Thus, we describe the histogram *H*_*t*_ by a heterogeneous two-component mixture model (Gaussian plus uniform) and estimate its parameters (*μ*,*σ*^2^ for the Gaussian, *ω*_G_ for the Gaussian mixture coefficient) by the EM algorithm, as outlined above. To start the EM iteration, we set *μ* to the location of the maximum of *H*_*t*_ and *σ*^2^:=1, while *ω*_G_ is immediately estimated in the E-step. After convergence, having estimated *μ* and *σ*, we say that all intensities up to *μ*+2*σ* belong to the baseline and adjust the chromatogram as follows: $S_{r,t}^{\prime }:=\max\{S_{r,t}-(\mu +2\sigma ),\;0\}$ for all *r*∈*R*. After repeating this step for every *t* with individually estimated *μ*(*t*), *σ*(*t*), the baseline *B*_*t*_=*μ*(*t*)+2*σ*(*t*) has been removed.

### De-noising (dn)

The goal of de-noising is to subtract a substantial amount of noise from the IMSC *S*(*r*,*t*) by estimating whether the intensity *S*(*r*,*t*) at coordinates (*r*,*t*) belongs to a peak region or can be explained by background noise. In previous work on de-noising Bader [13] uses a wavelet transform but applies it only spectrum wise. Our novel approach is similar to the Baseline Correction (bc) module in the sense that the EM algorithm is used, but the model is more complex and the subtraction works differently.

The method is not applied to *S* directly, but to the locally averaged

$$\begin{array}{c} A_{r,t}:=\frac{1}{{\left(2\rho +1\right)}^{2}}\cdot \overset{r+\rho }{\underset{r^{\prime }=r-\rho }{\sum }}\overset{t+\rho }{\underset{t^{\prime }=t-\rho }{\sum }}S_{r^{\prime },t^{\prime }}, \end{array}$$

where *ρ* is the smoothing_radius parameter. The spectra in our measurements consist of 2500 data points with a maximum reduced inverse mobility of *t*=1.45 Vs/cm^2^. With respect to tolerance *Δt*:=0.003 Vs/cm^2^ (value explained in Section ‘Modules for peak picking’) we obtain a tolerance of (2500·0.003)/1.45≈5 index units. We chose *ρ*=4 to avoid taking noise into consideration for smoothing.

Considering a histogram of all *A*-values also with bin size 1 (see Additional file 1: Supplement E), we identify three components: the noise component (the one of interest and to be removed) is modeled as a Gaussian distribution, the signal component (to be kept) is modeled as an Inverse Gaussian distribution and a background component (that explains every intensity not well explained by the other components) is modeled as a uniform distribution over all measured intensities. This yields a three-component heterogeneous mixture model (Gaussian plus Inverse Gaussian plus uniform), whose parameters are again estimated by the EM algorithm.

After convergence and a final E-step, we obtain the expected membership values *W*_*r*,*t*_ (which are a weighted normalized values of every probability computed by the probability models) of each data point (*r*,*t*) in the noise component. We adjust the original IMSC such that only the non-noise fraction remains, i.e., $S_{r,t}^{\prime }:=S_{r,t}\cdot (1-W_{r,t})$ for all *r*∈*R*,*t*∈*T*.

### Smoothing (s)

The smoothing module consists of two consecutive methods. The first method is a lowpass filter. The IMSC is transformed from the time/signal domain into the frequency/signal domain by a two-dimensional fast Fourier transform (2DFFT). All frequencies above a given frequency threshold (parameter fftcutoff) are removed, i.e., set to zero intensity. The inverse transformation of the filtered matrix is done using the inverse two-dimensional fast Fourier transform (I2DFFT).

The result is smoothed by a Savitzky-Golay filter (SGF) [14] on local windows using smoothing_radius*ρ*=4 (i.e., 9×9 data points) around each data point. To handle the boundaries of the measurement, we expand the data matrix with a margin containing only zero values. Since the data at the boundary of the measurement does not contain important data, this procedure is uncritical. The SGF computes a weighted average across the window.

## Modules for peak candidate detection

We discuss two modules to find peak candidates. Both use parameter *I* (intensity_threshold) as signal intensity threshold.

### Local maxima (lm)

This module reports a peak candidate for every local intensity maximum with intensity at least *I* in a surrounding area. To report a point (*r*,*t*), we require (1.) that (*r*,*t*) is a local maximum in the sense the each of its eight direct neighbors has a lower or equal signal intensity than *S*_*r*,*t*_ but of at least *I*, and (2.) that the contiguous area around (*r*,*t*) with signal intensity at least *I* is of sufficient size. In other words, we discard points where the surrounding high-intensity area size consists of too few points. The required number of points is controlled by a parameter *A*≥9 (area_size). (By the first condition, (*r*,*t*) and its eight neighbors always account for nine points; the parameter *A* can be used to impose stricter conditions.)

### Cross finding (cf)

The basic idea of Cross Finding is to find maxima based on the ideas by Fong *et al.*[15]. To avoid overlooking peaks at the borders, the matrix’s borders are temporarily padded by zeros.

We construct two auxiliary matrices *D*^R^ and *D*^T^, both with the same dimensions |*R*|×|*T*|. In *D*^T^, discrete derivatives of spectra are stored (partial derivatives with respect to reduced inverse mobility), $D_{r,t}^{T}:=S_{r,t+1}-S_{r,t}$; analogously derivatives of chromatograms are stored in *D*^R^. We describe how *D*^T^ is analysed.

In each derived spectrum (for fixed retention time *r*), we mark downward zero crossings; these are indices *t* with $D_{r,t-1}^{T}\geq 0$ and $D_{r,t}^{T}<0$. The resulting indices *t* are called *active positions* for retention time *r*.

While we scan through the spectra, we maintain two data structures. The first one is an *active set* containing lists of active positions connected across several spectra. The second one is a *finalized set*, where lists from the active sets are moved when they have been processed. Initially both sets are empty.

**Figure 5:**
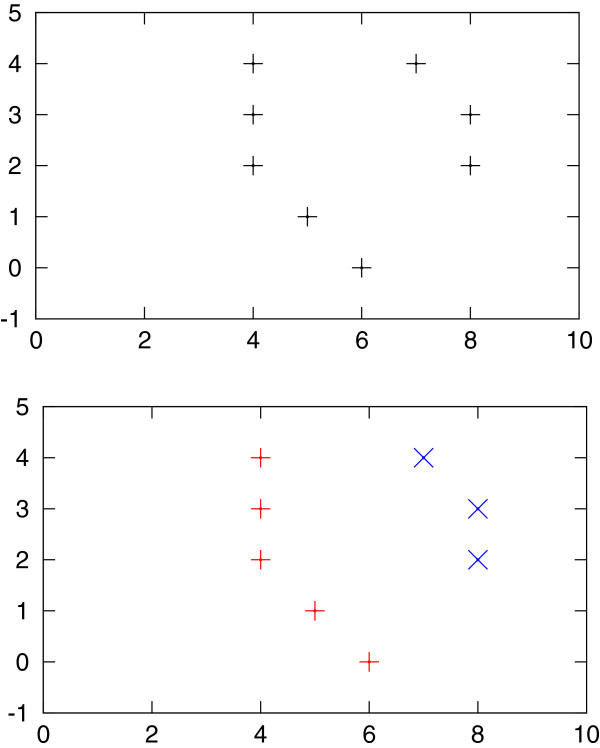
**Cross finding: Active positions (marking potential peak maxima) are initially unaligned (top) and then connected by alignment across spectra (bottom; shown as red + and blue x).** The same procedure is repeated over all chromatograms giving horizontal bands instead of vertical bands. Intersecting the results from both dimensions results in peak candidates.

We want to connect active positions between consecutive retention times, i.e., we want to find active positions for spectrum *r*+1 corresponding to active positions in spectrum *r* (see Figure 5(left)). To find the correspondences, we use a variant of global alignment between the two sorted integer lists *A* and *A*^+^ containing the active positions. The score of aligning *A*[ *i*] to *A*^+^[ *j*] depends on the distance between *A*[ *i*] and *A*^+^[ *j*]. We use the following score function: score(*i*,*j*):=(1+|*A*[ *i*]−*A*^+^[ *j*]|)^−1^∈[ 0,1]. To prevent that two positions with a high distance are aligned, we introduce a gap score *γ*=0.1. As we will thus never align positions *A*[ *i*] and *A*^+^[ *j*] with distance larger than 9 index units, we can solve the alignment problem very efficiently by only considering (*i*,*j*) with |*A*[ *i*]−*A*^+^[ *j*]|≤9.

Three scenarios are possible between the aligned position pairs: 

(1) If *A*^+^[ *j*] is not aligned to any *A*[ *i*], it is a “new” active position, and a new list, containing only *A*^+^[ *j*] is inserted into the active set.

(2) If *A*^+^[ *j*] is aligned to some *A*[ *i*], the corresponding list containing *A*[ *i*] is already in the active set and extended by *A*^+^[ *j*].

(3) Each *A*[ *i*] that is not aligned to any *A*^+^[ *j*] finalizes its corresponding active list, and the list is moved into the finalized set.

After processing all spectra and finalizing each remaining list, we obtain several position lists pointing out consecutive maxima throughout each spectrum; see Figure 5(right).

The same procedure is analogously performed with matrix *D*^R^. We report the intersection of positions found from both matrices (which can be visualised as crosses; hence the name “Cross Finding”). If more than one position overlap is found between two lists, the position with the highest signal is reported. Each reported point whose signal exceeds *I* is a candidate for a peak location.

## Modules for peak picking

The previous step, peak candidate detection, considers each potential peak location separately. Two peak candidates may be called close to each other, e.g., by detecting two local maxima of the same underlying peak that arise because of noise in the data.

Thus, not every peak candidate corresponds to a VOC from the breath sample, and the purpose of peak picking is to thin out the candidate list.

Bödeker [16] introduced a minimum distance in retention time and in reduced inverse mobility such that two peaks exceeding those distances belong to distinct compounds. We write *Δt* for the necessary distance in reduced inverse mobility and *Δr* for that in retention time. We use a constant *Δt*:=0.003 Vs/cm^2^ (tol_rim) for the reduced inverse mobility. In retention time we use an affine-linear *Δr*:=*p*·*r*+*c* for a peak at position (*r*,*t*), where *c*:=3 s (tol_rt) and *p*:=0.1 (tol_percent_rt), as suggested by Hauschild *et al.*[9]. We now describe three modules for peak picking.

### Merging by signal intensity (ms)

We sort the *n* peak candidates by descending signal intensity into a list [ (*r*_*i*_,*t*_*i*_,*s*_*i*_)]_*i*=1,…,*n*_ with *s*_1_≥*s*_2_≥⋯≥*s*_*n*_, resolving ties arbitrarily. First we mark each candidate as unmerged. Iterating the list, we skip merged candidates and report each unmerged candidate we encounter. When this happens for candidate (*r*_*i*_,*t*_*i*_,*s*_*i*_), we find which peaks fall into the box (*r*_*i*_±*Δr*)×(*t*_*i*_±*Δt*) and mark them as merged, and continue iterating through the list. In this way, we greedily pick peaks with highest signal as representative for all peaks in the surrounding box. This method [16] was used by Hauschild *et al.*[9].

### Cluster editing (ce)

We find clusters of peaks, from which we pick a representative (peak with highest signal), by solving an instance of the weighted cluster editing problem [17,18]: Let *G*=(*V*,*E*) be a weighted, undirected graph without loops with a symmetric similarity weight function w:V2→ℝ, such that *E*={{*u*,*v*}:*w*(*u*,*v*)≥0}. The graph can be modified by adding a non-existing or removing an existing edge {*u*,*v*}, which incurs a cost of |*w*(*u*,*v*)|. The costs for several modifications are added to yield the total cost. The objective is to find a set of edge modifications with minimum cost such that the resulting graph consists of disjoints cliques (i.e., is transitive).

Every candidate peak is a vertex *u*=(*r*_*u*_,*t*_*u*_). The similarity *w*(*u*,*v*) between two vertices *u*,*v* depends on their distances on the *r*- and *t*-axis. We use the distance measure

$$d^{2}(u,v):=\frac{1}{2}\left[\right)\left(\right)\frac{t_{u}-t_{v}}{\mathrm{\Delta t}}{))}^{2}+\left(\right)\frac{r_{u}-r_{v}}{\mathrm{\Delta r}}{))}^{2}])$$

and the similarity weight function (with a constant scaling factor *b*)

$$w(u,v):=\left\{\begin{array}{ll} 2^{b(1-d^{2}(u,v))}-1\; & \text{if}\;\;d^{2}(u,v)\leq 1,\\ 1-d^{2}(u,v)\; & \text{otherwise.} \end{array}\right)$$

The range for *w*(*u*,*v*) is therefore [ −*∞*, 2^*b*^−1]. If the distance between the two candidates *u* and *v* is zero, then the edge (*u*,*v*) has the maximum weight 2^*b*^−1. If the *t*- and *r*-distances of *u* and *v* are equal to *Δt* and *Δr*, respectively, then *d*^2^(*u*,*v*)=1 and *w*(*u*,*v*)=0. For larger distances, the weights are negative. Parameter *b* called ce_weight_exponent can be set by the user.

The weighted cluster editing problem is solved with the *yoshiko 2.0* software (http://www.cwi.nl/research/planet-lisa).

### EM clustering (emc)

This module uses the EM algorithm once again. Initially, each peak candidate represents a component. During the course of the algorithm, components can be merged. The remaining components will represent the picked peaks.

Each component is a two-dimensional Gaussian distribution with independent dimensions, i.e., diagonal covariance matrix. Initially, the mean of every component is the (*r*,*t*) location of the corresponding peak candidate. The standard deviation on the *r*- and *t*-axis is set to *Δr*/3 and *Δt*/3, respectively, since 6*σ* covers most of a Gaussian bell curve. In the E-step, the hidden membership coefficients of each peak to each component are estimated. When a peak candidate is close to another one, the probability that the first model also (partially) describes the second candidate is comparatively high. In the maximization phase, the parameters of each component are re-estimated based on candidate membership using maximum likelihood estimators. In the case of two close candidates, the mean of both components moves towards their middle. When the distance between the means of two components drops below a given threshold, the components are merged: The component of the candidate with lower signal is removed, and its weight is added to the remaining model. The E- and M-steps are repeated until convergence.

When updating the variance by maximum likelihood estimation, we must be aware that the variance of a component described by only one peak tends to zero, which leads to a singularity in the Gaussian density function. Therefore, we restrict the estimated standard deviation to values above the threshold *τ*:=10^−5^ Vs/cm^2^.

## A module for peak modeling (pme)

Peak modeling is an optional step that estimates a parametric model of a peak shape. We have so far implemented one module (simply called Peak Model Estimation (pme)) using shifted Inverse Gaussian distributions, consider [12]. If it is not desired to model the peaks, the empty *ε* module (e) can be used instead. It outputs the peak list without any modification.

A whole IMSC is interpreted as a sample from a mixture model of different shifted Inverse Gaussians plus a uniform background noise model. Each component (peak) can then be described by seven parameters (three for both shifted Inverse Gaussians in both *r*- and *t*-dimension, plus one mixture coefficient). The challenge is to estimate the parameters correctly, especially when peaks overlap. Again, the EM algorithm is utilized for this purpose.

For efficiency, each component model is evaluated only in a surrounding box enclosing the peak. Starting from the picked peak location, the box borders are expanded in all four main directions until the signal intensity drops to zero in each direction. The parameter expansion_size determines how much the box around the peak is expanded additionally.

When two boxes intersect, both boxes are merged into their convex hull. After that process we have a set of boxes containing at least one peak. Now we can apply EM to each box independently, with the advantage of processing smaller boxes in contrast to the whole signal matrix. Starting parameters for each component are estimated from the locations of picked peaks and additional assumptions: The parameters are chosen such that their modes correspond to the known (*r*,*t*) values, the mean is set slightly higher (*μ*=mode+10^−3^ index units), and the standard deviation is set to 1 index unit in both dimensions. As the model parameters have a rather technical interpretation, they are translated back into mode, mean and standard deviation of the distribution, which are conveniently compared and interpreted.

## Architecture and implementation

The framework consists of a number of classes representing input, output and parameters and, importantly, four function interfaces, one for each major step of a pipeline. The steps have unified interfaces to guarantee the modularity of the framework and the exchangeability of the modules with future ones.

### Input

The standardized .csv format for MCC/IMS measurements is described by Vautz *et al.*[19,20]. A more efficient binary format (.ims) has also been developed internally. An abstract class IMSFile provides the interface for loading and storing those formats, and the classes IMSFileCSV and IMSFileIMS instantiate the interface for the respective format.

The class IMSMeasurement stores a sequence of retention time points *R*, a sequence of drift time points and (proportionally) reduced inverse mobilities *T*. Additionally, a measurement_parameters map stores all meta information e.g. date, time, name or various sample information of a measurement. It also stores an IMSMatrix that represents an IMSC (*S*_*r*,*t*_), i.e., it contains the raw intensity values as a matrix.

### Output

The class IMSPeak describes a single peak. It stores the name of the originating measurement, its peak name (ID), retention time and reduced inverse mobility of the peaks mode, the signal intensity and the volume (if not calculated, equal to the intensity). The indices of both retention time and reduced inverse mobility are also stored. A map peak_parameters may store additional parameters, e.g., parameters estimated for inverse Gaussian distributions, as described in Section ‘A module for peak modeling (pme)’.

The class IMSPeakList stores the resulting list of such peaks found by the candidate detection, picking and modeling steps. Every IMSPeakList contains a list named parameter_names, which stores the names of additional parameters for every peak. These are the keys for the above mentioned peak_parameters map.

The output format is a .csv file with one line per peak containing the peak’s measurement name, peak name, retention time, reduced inverse mobility, signal, volume, retention time index, reduced inverse mobility index and additional parameters.

### Module parameters

A map called parameter_map stores all peak extraction parameters (Table 1).

**Table 1:** Parameters used for evaluation

**Modules**	**Parameter name**	**Value**
s	fftcutoff	500
s, dn	smoothing_radius	4
lm	area_size	9
lm, cf	intensity_threshold	{5, 10, 15}
ce	ce_weight_exponent	26
All picking	tol_rt	3
All picking	tol_rt_percent	0.1
All picking	tol_rim	0.003
pme	expansion_size	10

All computations were repeated for three different values of **intensity_threshold**.

### Function interfaces

Using unified function interfaces for each step ensures the modularity of the pipeline. A preprocessing function takes an IMSMeasurement and a parameter_map manipulating the provided matrix.

A candidate detection method requires an IMSMeasurement and a parameter_map and returns an IMSPeakList. The picking functions take those results as input and return an IMSPeakList that contain a subset of the input list. Finally the modeling step requires again the IMSMeasurement in addition to an IMSPeakList and the parameter_map. This step returns an IMSPeakList of the same size as the input one. To augment a particular step with a new module, these interfaces must be used.

## Results

### Dataset

We tested the framework on a dataset of 69 measurements, of which 39 are from different patients suffering from the same disease and 30 from a control group not showing corresponding symptoms. The disease is known but irrelevant with respect to this article and cannot be disclosed due to confidentiality agreements within the clinical study approved by the state ethics committee. Our dataset has been anonymized and serves as an illustration of the framework. For every of the 69 measurements, a manually annotated peak list was provided by an expert annotator.

### Evaluation of pipelines

By combining the implemented modules, we obtain 108 individual pipelines. We name the pipelines by concatenating the shortcuts of the used modules in order. For example, the pipeline using (in that order) the modules Smoothing, De-Noising, Baseline Correction, Local Maxima, EM Clustering and No Modeling is named s-dn-bc-lm-emc-e. There are not 144 pipelines because of redundancy between pipelines using the empty module as fourth step and those using it as third one (consider the example before and s-dn-bc-lm-e-emc, which is the same computational pipeline). We used the parameters shown in Table 1.

To evaluate each pipeline, we compare the final obtained peak list with one that was manually annotated by an expert MCC/IMS development engineer. For the comparison, we considered only peaks with a retention time above 5 s and an inverse reduced mobility above 0.48 Vs/cm^2^, as is standard practice.

Agreement of an automatically obtained peak list with that obtained by a domain expert is generally considered favourable. However, one should be aware that manually annotated list may also be incomplete or contain extraneous peaks. Nevertheless considering the manually annotated peaks ground truth, we compute the following quantities. Peaks detected by both methods, manual and automatic, within one measurement are true positives (TP). (Below, we address the question when peaks with slightly different location parameters should be considered the same.) Accordingly, manually annotated peaks that are not detected by the pipeline are false negatives (FN) and automatically detected peaks not found in the manual annotation are false positives (FP). We compute the sensitivity SENS:=TP/(TP+FN) and the positive predictive value PPV:=TP/(TP+FP). Their geometric mean $G:=\sqrt{\text{SENS}\cdot \text{PPV}}$ summarizes both measures, which is referred to as Fowlkes-Mallows index [21]. Further, the Jaccard index between two peak lists is *J*:= TP / (FN + TP + FP) ∈[ 0,1]. From this, we derive a distance measure *d*:=1/*J*−1∈[ 0,*∞*]. The distance and geometric mean are calculated separately for each combination of pipeline and measurement. To determine these quantities for a particular pipeline, we average over all measurements of the dataset. It remains to define what it means that “the same” peak has been detected by both methods, since the location parameters (*r*,*t*) may differ slightly. All peak picking modules can be used for this decision, and we chose “Merging by Signal Intensity” (ms). Imagine a box around every manually annotated peak (*r*,*t*) of widths 2*Δr* and 2*Δt*, respectively. Then we successively count each box containing at least one automatically found peak and delete it. In case of two or more peaks within the box we count the closest one.

### Ranking the pipelines

**Figure 6:**
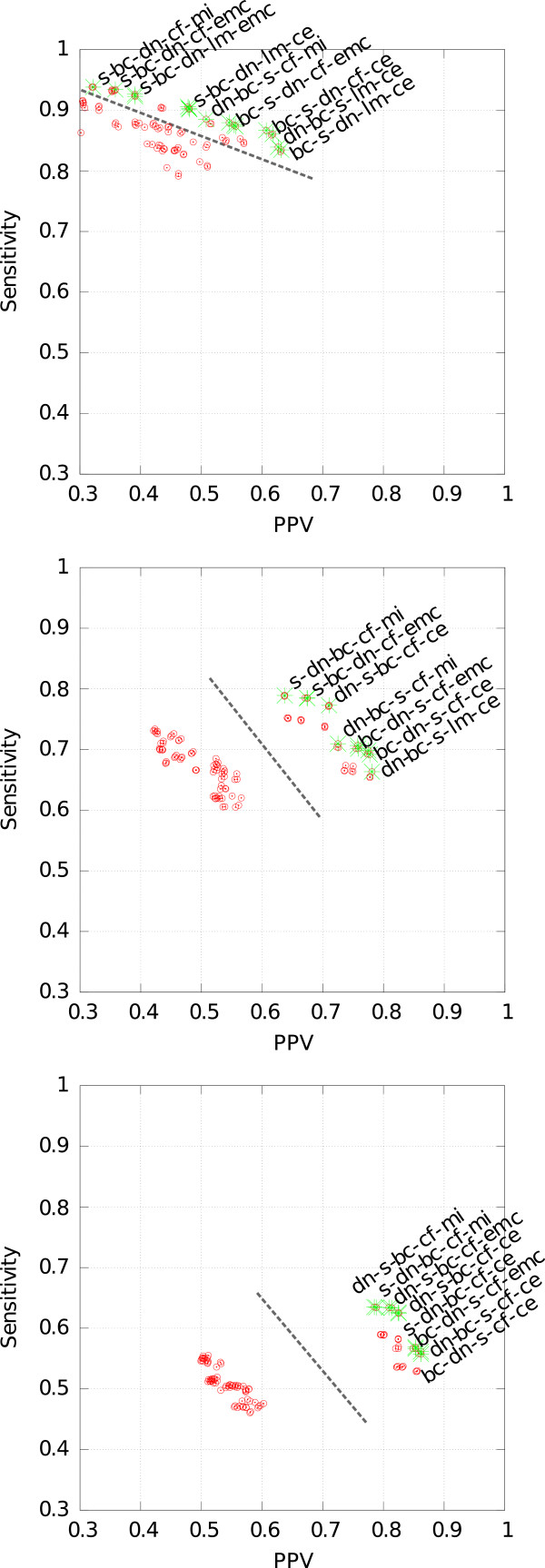
**Comparing the results of all pipelines with the manually picked peaks according to sensitivity and PPV.** The green crosses indicate the Pareto front. Top, middle and bottom figure correspond to signal intensity thresholds *I*=5,10,15, respectively. The dashed lines separate two clusters of pipelines.

Figure 6 shows a plot of SENS against PPV for each pipeline for different parameter values of the signal intensity threshold *I*. The Pareto front is visualized in each plot. Considering only pipelines that are Pareto-optimal, we rank the ten best ones by their geometric mean and distance separately. The results of the rankings are presented in Tables 2, 3 and 4 for signal intensity thresholds *I*∈{5,10,15}, respectively. Note that each of these tables reports two rankings, the first two columns for the geometric mean *G* and the both remaining columns for the distance *d*.

**Table 2:** **Pipelines of the Pareto front for signal threshold *I = 5***

**Pipeline**	***G***	***d***	**Pipeline**
bc-s -dn-cf-ce	0.729	0.850	bc-s -dn-cf-ce
bc-s -dn-lm-ce	0.726	0.853	bc-s -dn-lm-ce
dn-bc-s -cf-ce	0.725	0.855	bc-dn-s -cf-ce
dn-bc-s -lm-ce	0.725	0.861	dn-bc-s -lm-ce
bc-s -dn-cf-emc	0.697	0.862	bc-dn-s -lm-ce
bc-dn-s -cf-emc	0.697	0.868	dn-bc-s -cf-ce
dn-bc-s -cf-emc	0.693	1.012	bc-s -dn-cf-emc
dn-bc-s -cf-mi	0.671	1.016	bc-s -dn-lm-emc
s -dn-bc-lm-ce	0.658	1.018	bc-dn-s -cf-emc
dn-s -bc-lm-ce	0.658	1.021	bc-dn-s -lm-emc

**Table 3:** **Pipelines of the Pareto front for signal threshold** ***I=10***

**Pipeline**	***G***	***d***	**Pipeline**
dn-s -bc-cf-ce	0.741	0.776	dn-s -bc-cf-ce
dn-bc-s -cf-ce	0.736	0.783	s -dn-bc-cf-ce
bc-dn-s -cf-ce	0.733	0.785	s -bc-dn-cf-ce
dn-bc-s -cf-emc	0.732	0.811	dn-bc-s -cf-ce
bc-dn-s -cf-emc	0.729	0.822	bc-dn-s -cf-ce
s -bc-dn-cf-emc	0.728	0.825	dn-bc-s -cf-emc
s -dn-bc-cf-emc	0.727	0.827	bc-s -dn-cf-ce
dn-bc-s -lm-ce	0.720	0.836	bc-dn-s -cf-emc
dn-bc-s -cf-mi	0.717	0.838	s -bc-dn-cf-emc
s -dn-bc-cf-mi	0.709	0.839	s -dn-bc-cf-emc

**Table 4:** **Pipelines of the Pareto front for signal threshold** ***I=15***

**Pipeline**	***G***	***d***	**Pipeline**
dn-s -bc-cf-ce	0.718	0.916	dn-s -bc-cf-ce
s -dn-bc-cf-ce	0.718	0.916	s -dn-bc-cf-emc
s -dn-bc-cf-emc	0.718	0.916	s -bc-dn-cf-emc
dn-s -bc-cf-emc	0.716	0.921	dn-s -bc-cf-emc
s -dn-bc-cf-mi	0.707	0.923	s -dn-bc-cf-ce
dn-s -bc-cf-mi	0.706	0.923	s -bc-dn-cf-ce
dn-bc-s -cf-emc	0.697	0.959	s -dn-bc-cf-mi
dn-bc-s -cf-ce	0.696	0.959	s -bc-dn-cf-mi
bc-dn-s -cf-emc	0.695	0.962	dn-s -bc-cf-mi
bc-dn-s -cf-ce	0.693	1.045	dn-bc-s -cf-emc

We find that, for the candidate detection step, almost all Pareto-optimal pipelines use Cross Finding (cf). The picking step is best done by Cluster Editing (ce). For every signal intensity threshold *I*, the pipelines split into two groups. The first group has both relatively low sensitivity and low positive predictive value, while the second one has high values for both measures. We note that the pipelines of the first group are characterized by the utilization of the pme module (see Additional file 1: Supplements A, B and C). By modeling, the peak coordinate moves slightly, yielding larger average differences to the manual annotation based on grid coordinates. So module pme seems to be unnecessary. However, the volume of a peak may contain important information (not evaluated here), and we cannot infer it only from the position and intensity at those coordinates alone. Concerning the threshold *I*, the choice of 10 yields the best results. For both *I*=10 and 15, the best pipeline is dn-s-bc-cf-ce. The top values in Table 3 range around 0.70 for the geometric mean. We note that individual measurement properties (high or low noise, characteristic VOCs, etc.) were not taken into consideration for choosing the module parameters. An additional step of parameter estimation from global measurement properties would likely improve the results.

## Discussion and conclusion

We presented the first framework for fully automatic processing of MCC/IM measurements, consisting of different modules for four distinct computational steps. The presented framework processes a single IM measurement and outputs a peak list within a few seconds. A domain expert, who before had the time consuming task of peak annotation, may now quickly verify the resulting automatically generated peak list and manually reject a few potential false positives. In practice, higher sensitivity of the automated pipeline (at the cost of more false positive predictions) may be desirable, as this type of error can be better compensated by statistical learning methods during classification than a false negative rate.

The best pipeline achieves a geometric mean of sensitivity and positive predictive value of 0.741 when compared to a manual expert manual annotation, without tuning the parameters for the single modules. Since the manually extracted peaks are annotated by one single expert, one cannot be certain whether that solution is fully correct; some of the peaks evaluated as false positives in our pipelines might in fact be false negatives of the expert, but this is difficult to quantify, except on a case-by-case basis.

Hauschild *et al.*[9] discovered that the hand picking method by domain experts yields the best results for classification compared to automatic peak picking methods (e.g. IPHEx [22] or Visual Now [23]). Thus it is reasonable to compete with a domain expert.

The state of the art today is that every expert has his own “manual procedure” for peak extraction, based on certain human-observable features of the visualized data matrix. We observed two experts and attempted to infer their “internal algorithm” and express this knowledge as our parameters. The most significant parameter values were determined by domain experts’ experiences, i.e. the tolerances used for the picking step. The intensity threshold from the candidate detection step was tested empirically. We reported on three different values (Section ‘Results’). For the other parameters we used values selected by our own experience. These last parameters do not influence the results as much as the previously mentioned ones.

### Future work

Our future work will consist in the effort to estimate as many of the algorithms’ parameters as possible from the given data matrix. The tolerance parameters of the picking step were so far determined by domain experts’ experiences, and it will be difficult to learn them purely from the data. For some other parameters it may be possible, e.g. for the signal intensity by determining the noise level of the background noise. Also, for the area size of the peak candidate detection step and the expansion size for the peak modeling we see a possibility to automatically determine the values.

The framework with the modules described in this article are available at http://www.rahmannlab.de/research/ims, as well as the anonymized datasets and Additional file 1: Supplement.

## Competing interests

We declare that we have no competing financial interests. We do declare that JIB is chairman of a company, B & S Analytik GmbH that builds and sells ion mobility spectrometers. However, our algorithms are general-purpose and not restricted to their instruments.

## Authors’ contributions

MDA and DK developed and implemented the framework including every presented module. MDA, DK and SR drafted the manuscript and JIBB provided the data for evaluation. All authors read and approved the final manuscript.

## Supplementary Material

Additional file 1 Supplement. Click here for file
