# Supplementary material for: A modular computational framework for automated peak extraction from ion mobility spectra
Source: BMC Bioinformatics. 2014 Jan 22;15:25. doi: 10.1186/1471-2105-15-25 (PMC3930762; doi:10.1186/1471-2105-15-25)

Supplement A:
This table shows the results of comparing all possible pipeline instances for an intensity threshold I = 5. The instances are sorted by their geometric mean. The highlighting indicates the division into the two groups. The rows with blue characters contain the pareto-optimal instances.

Pipeline instance	Average PPV	Average SENS	Geometric Mean
bc-s-dn-cf-ce-e	0.6176960434782611	0.8604853333333333	0.7290530747970773
bc-dn-s-cf-ce-e	0.6157173623188407	0.859032420289855	0.7272696721074235
bc-s-dn-lm-ce-e	0.6317268260869565	0.8346429420289856	0.7261310740381868
dn-bc-s-cf-ce-e	0.6069900579710146	0.8666766956521736	0.725302790382086
dn-bc-s-lm-ce-e	0.6266765942028986	0.8392571594202899	0.7252191519298883
bc-dn-s-lm-ce-e	0.6306465072463769	0.8316906956521741	0.7242256777568392
bc-s-dn-cf-emc-e	0.5565238695652173	0.8734348840579708	0.6971996568338146
bc-dn-s-cf-emc-e	0.5541914202898549	0.8753877536231881	0.696514452459373
bc-s-dn-lm-emc-e	0.5697321304347825	0.8473133043478258	0.6947960952911452
bc-dn-s-lm-emc-e	0.5700628115942029	0.8450512028985506	0.6940693514811117
dn-bc-s-lm-emc-e	0.5640943768115941	0.8526521739130434	0.6935245465594777
dn-bc-s-cf-emc-e	0.545966623188406	0.8795365072463766	0.6929628971541184
bc-s-dn-lm-mi-e	0.541259797101449	0.8502833333333331	0.678398249171343
bc-dn-s-lm-mi-e	0.5400517391304345	0.8480212318840579	0.6767387539505322
dn-bc-s-lm-mi-e	0.534677768115942	0.8548975652173914	0.6760878065297345
bc-s-dn-cf-mi-e	0.5163679855072463	0.8776414057971013	0.6731908531086833
bc-dn-s-cf-mi-e	0.5144090579710145	0.8776414057971013	0.6719127092059234
dn-bc-s-cf-mi-e	0.5079521594202899	0.8852256666666665	0.6705611746571666
s-dn-bc-lm-ce-e	0.47980527536231893	0.902816144927536	0.658161035779621
dn-s-bc-lm-ce-e	0.4781167246376812	0.9055797971014492	0.6580067222211341
s-bc-dn-lm-ce-e	0.4801988695652174	0.9016084057971012	0.6579903777824306
bc-s-dn-cf-ce-pme	0.509563043478261	0.8431052318840577	0.6554504313304595
bc-dn-s-cf-ce-pme	0.5078424492753623	0.8389181304347822	0.652716047069097
dn-bc-s-cf-ce-pme	0.48674671014492754	0.8474889999999998	0.6422713465771414
bc-s-dn-lm-ce-pme	0.5098788550724638	0.8087069275362317	0.6421390521540827
bc-dn-s-lm-ce-pme	0.5102337681159421	0.8064519275362316	0.6414662935736968
dn-bc-s-lm-ce-pme	0.4973611159420291	0.814964376811594	0.6366565729685154
bc-s-dn-cf-emc-pme	0.46782788405797104	0.8642639565217387	0.6358669499566205
bc-dn-s-cf-emc-pme	0.46525840579710154	0.8619602753623188	0.6332726613201481
s-bc-dn-cf-ce-e	0.43643753623188397	0.903373376811594	0.627906084437099
dn-s-bc-cf-ce-e	0.43397611594202895	0.9047641884057971	0.6266147527211515
s-dn-bc-cf-ce-e	0.43359407246376797	0.903373376811594	0.6258572851753705
bc-s-dn-lm-emc-pme	0.4710657391304349	0.8278785072463766	0.6244879509856179
bc-s-dn-cf-mi-pme	0.4462829275362318	0.8728652898550725	0.6241353033608044
bc-dn-s-lm-emc-pme	0.4705888695652174	0.8264654347826085	0.623638865601779
bc-dn-s-cf-mi-pme	0.4449836086956522	0.8717246666666667	0.6228187440679254
bc-s-dn-cf-pme-ce	0.4618610869565217	0.8382757101449275	0.6222273946531014
dn-bc-s-cf-emc-pme	0.44557134782608676	0.8641328115942026	0.6205101301048797
bc-dn-s-cf-pme-ce	0.46022707246376804	0.8350668985507246	0.6199357983141467
dn-bc-s-lm-emc-pme	0.4568176956521739	0.8331713188405795	0.6169338716233986
bc-s-dn-lm-mi-pme	0.4557026376811594	0.8342234347826084	0.6165693956448628
bc-dn-s-lm-mi-pme	0.4551620579710145	0.8330864202898548	0.6157835086512433
bc-s-dn-cf-pme-emc	0.4315679130434782	0.8711050434782608	0.6131402658899197
bc-dn-s-cf-pme-emc	0.42879442028985487	0.8694146666666664	0.610573630273066
dn-bc-s-cf-mi-pme	0.424475	0.8733673623188402	0.6088699459821364
bc-s-dn-cf-pme-mi	0.42100542028985505	0.8784371594202898	0.608133871363835
bc-dn-s-cf-pme-mi	0.420306420289855	0.8761538985507245	0.606838618351581
bc-s-dn-lm-pme-ce	0.4624493913043477	0.7953569420289852	0.6064753364408083
dn-bc-s-lm-mi-pme	0.4392756956521738	0.8358601449275361	0.605948056050162
bc-s-dn-lm-pme-emc	0.438114768115942	0.836406333333333	0.6053445025595248

bc-dn-s-lm-pme-ce	0.4622387681159419	0.7913618550724634	0.6048124742617682
dn-bc-s-cf-pme-ce	0.43311859420289844	0.8414643768115943	0.6037001473881348
bc-dn-s-lm-pme-emc	0.43608920289855074	0.8328279999999997	0.6026502291309562
bc-s-dn-lm-pme-mi	0.43034401449275367	0.8426196231884056	0.602176312497652
dn-s-bc-lm-emc-e	0.39070185507246374	0.9262613478260869	0.6015746228669996
s-bc-dn-lm-emc-e	0.39096891304347814	0.9228208260869565	0.6006615147561079
s-dn-bc-lm-emc-e	0.39025563768115934	0.9240285652173913	0.6005059175016677
bc-dn-s-lm-pme-mi	0.42722905797101446	0.8372433188405795	0.5980758099110735
dn-bc-s-lm-pme-ce	0.4433286956521739	0.8052795072463765	0.5974977101068768
dn-bc-s-cf-pme-emc	0.40669208695652165	0.8718543623188404	0.5954630720150466
dn-bc-s-lm-pme-emc	0.4190562463768116	0.8435640579710144	0.5945593222814056
dn-bc-s-cf-pme-mi	0.3975651884057971	0.8787484057971015	0.5910666421919093
dn-bc-s-lm-pme-mi	0.41099834782608685	0.8445302318840578	0.5891523826596391
dn-s-bc-lm-ce-pme	0.3907684347826087	0.880539072463768	0.5865891876872575
s-bc-dn-lm-ce-pme	0.3922819275362318	0.8749596956521736	0.5858590922115567
s-dn-bc-lm-ce-pme	0.39064875362318835	0.8769303333333331	0.5852962854239157
s-bc-dn-cf-emc-e	0.35835798550724635	0.9342342318840579	0.5786106612653111
s-dn-bc-cf-emc-e	0.3578441449275362	0.9324892753623187	0.5776554573412483
dn-s-bc-cf-emc-e	0.3558369275362318	0.9320269420289854	0.5758902703055364
dn-s-bc-lm-mi-e	0.35253778260869545	0.9332895362318839	0.5736024961897273
s-bc-dn-lm-mi-e	0.35317040579710135	0.9305391449275362	0.5732703440996627
s-dn-bc-lm-mi-e	0.35260814492753617	0.9317468840579709	0.5731854327612428
s-bc-dn-cf-ce-pme	0.3632614782608695	0.872475217391304	0.562971257894698
dn-s-bc-cf-ce-pme	0.35905143478260865	0.8791132608695649	0.5618245968731121
s-dn-bc-cf-ce-pme	0.35772672463768107	0.8766074057971011	0.559987407062812
s-bc-dn-cf-mi-e	0.32200684057971013	0.9380425652173913	0.5495963270937487
dn-s-bc-lm-emc-pme	0.3316050579710144	0.9066264637681158	0.5483082354623919
dn-s-bc-cf-mi-e	0.32044869565217377	0.9380425652173913	0.5482650057135985
s-dn-bc-cf-mi-e	0.3199180144927536	0.9380425652173913	0.547810838678861
s-dn-bc-lm-emc-pme	0.3305389130434782	0.9051735507246376	0.5469872773403669
s-bc-dn-lm-emc-pme	0.33146153623188396	0.8996820144927534	0.5460860579102567
dn-s-bc-lm-mi-pme	0.3047235942028985	0.9158139565217389	0.5282708779144287
s-dn-bc-cf-emc-pme	0.30579639130434794	0.9122279710144925	0.5281628741052509
s-bc-dn-cf-emc-pme	0.3080822898550725	0.9040346521739129	0.527747160816686
s-dn-bc-lm-mi-pme	0.3043318985507246	0.9147162753623187	0.5276147654456439
dn-s-bc-cf-emc-pme	0.3049956086956522	0.911538376811594	0.5272714690603872
s-bc-dn-lm-mi-pme	0.3052913188405797	0.9086879565217392	0.5267015707790068
s-bc-dn-lm-pme-ce	0.30170811594202895	0.8628782028985503	0.5102326497627974
s-bc-dn-cf-mi-pme	0.2831751014492753	0.9154242463768114	0.5091417816648718
dn-s-bc-cf-mi-pme	0.2811639420289855	0.9218212028985504	0.5091000719434812
s-dn-bc-cf-mi-pme	0.2797533188405797	0.9228175507246373	0.5080957316289444
s-dn-bc-lm-pme-ce	0.2946441014492752	0.8621606956521735	0.5040144476851003
dn-s-bc-lm-pme-ce	0.29439097101449274	0.8605661159420286	0.5033317936454484
s-bc-dn-lm-pme-emc	0.27387733333333325	0.9121851594202897	0.49982680897313864
s-bc-dn-cf-pme-ce	0.2858092463768117	0.866173985507246	0.497554553822016
dn-s-bc-lm-pme-emc	0.2705918260869566	0.9131495217391301	0.4970822835082963
s-dn-bc-lm-pme-emc	0.27054411594202904	0.9132564057971012	0.4970675476580338
dn-s-bc-cf-pme-ce	0.27904631884057973	0.8735604782608694	0.49372445325638054
s-bc-dn-lm-pme-mi	0.2651809130434782	0.9187077101449272	0.4935825659363537
s-bc-dn-cf-pme-emc	0.26284226086956525	0.9267448695652172	0.49354606346904645
s-dn-bc-cf-pme-ce	0.27785559420289857	0.8763877971014491	0.49346656636067293
dn-s-bc-lm-pme-mi	0.2619452028985508	0.9200533913043475	0.4909211466852055
s-dn-bc-lm-pme-mi	0.261612231884058	0.9201602753623185	0.4906375274360753
s-bc-dn-cf-pme-mi	0.2540484057971014	0.9315754057971015	0.4864825245808337
dn-s-bc-cf-pme-emc	0.25596384057971	0.9223495942028984	0.485889024870197
s-dn-bc-cf-pme-emc	0.2550640289855072	0.9245628260869562	0.4856158146847833
dn-s-bc-cf-pme-mi	0.24827839130434776	0.9285086376811593	0.48013397179919454
s-dn-bc-cf-pme-mi	0.24687220289855064	0.9299972318840578	0.4791559926837726

Supplement B:
This table shows the results of comparing all possible pipeline instances for an intensity threshold I = 10. The instances are sorted by their geometric mean. The highlighting indicates the division into the two groups. The rows with blue characters contain the pareto-optimal instances.

Pipeline instance	Average PPV	Average SENS	Geometric Mean
dn-s-bc-cf-ce-e	0.7103242753623186	0.7729500579710145	0.7409758361913855
s-dn-bc-cf-ce-e	0.709732724637681	0.7722254202898551	0.7403199656748406
s-bc-dn-cf-ce-e	0.7093704057971013	0.7709078985507247	0.7394993230741447
dn-bc-s-cf-ce-e	0.7749650144927536	0.6985806956521737	0.7357823040345866
bc-dn-s-cf-ce-e	0.7773827101449273	0.6918868985507245	0.7333900137778879
bc-s-dn-cf-ce-e	0.7734487826086955	0.6925770289855071	0.7318967549672479
dn-bc-s-cf-emc-e	0.7575695507246375	0.7067646086956519	0.7317262787939485
bc-dn-s-cf-emc-e	0.7581978695652172	0.7016134637681156	0.7293571371332827
bc-s-dn-cf-emc-e	0.7549392463768114	0.7016134637681156	0.727788114484515
s-bc-dn-cf-emc-e	0.6745526376811591	0.7846665797101448	0.7275293197141485
s-dn-bc-cf-emc-e	0.6739757826086956	0.7851789420289854	0.727455560114742
dn-s-bc-cf-emc-e	0.6707723188405796	0.7851789420289854	0.7257246720689435
dn-s-bc-lm-ce-e	0.7030166956521737	0.7390303333333331	0.7207986285549691
s-dn-bc-lm-ce-e	0.7034992898550723	0.7376814492753621	0.7203876565464442
dn-bc-s-lm-ce-e	0.7808161594202896	0.6634248115942027	0.7197310702290923
s-bc-dn-lm-ce-e	0.7031542173913042	0.7363639275362317	0.7195675098153932
dn-bc-s-cf-mi-e	0.7249859275362318	0.7096019999999998	0.7172527198634837
bc-dn-s-cf-mi-e	0.7257957826086957	0.7035983333333331	0.7146108752208019
bc-s-dn-lm-ce-e	0.7776939130434778	0.6553891304347824	0.7139272633917185
bc-s-dn-cf-mi-e	0.7233317826086957	0.7041159420289852	0.7136591900277239
bc-dn-s-lm-ce-e	0.7776000289855068	0.65389384057971	0.7130693300011065
dn-bc-s-lm-emc-e	0.7499770289855069	0.6724669999999997	0.7101653347994371
s-dn-bc-cf-mi-e	0.6372802753623189	0.7892148405797099	0.7091904193689211
dn-s-bc-cf-mi-e	0.636681043478261	0.7892148405797099	0.7088569166121038
s-bc-dn-cf-mi-e	0.6368988840579711	0.7878973188405796	0.7083861398431174
s-dn-bc-lm-emc-e	0.6644928985507244	0.7487051159420288	0.7053433438064578
bc-dn-s-lm-emc-e	0.7496316666666663	0.6636261594202897	0.7053192071181889
dn-s-bc-lm-emc-e	0.663612333333333	0.7493952463768114	0.7052006296345912
dn-bc-s-lm-mi-e	0.7376362898550722	0.6737272318840577	0.7049579105884863
bc-s-dn-lm-emc-e	0.7475165362318837	0.6644313188405796	0.7047505927799511
s-bc-dn-lm-emc-e	0.6641199275362317	0.7473875942028985	0.7045246588328243
bc-s-dn-lm-mi-e	0.7354452608695651	0.6656915507246375	0.6996997185802967
bc-dn-s-lm-mi-e	0.7355813333333331	0.6648863913043477	0.6993411315165441
s-dn-bc-lm-mi-e	0.6430048115942026	0.7522234057971012	0.6954734138134357
s-bc-dn-lm-mi-e	0.642625913043478	0.7509058840579708	0.6946593261106292
dn-s-bc-lm-mi-e	0.6413434492753621	0.7522234057971012	0.6945743687320842
dn-bc-s-cf-ce-pme	0.5581850724637683	0.6600641594202895	0.6069909065684185
bc-dn-s-cf-ce-pme	0.5586906811594203	0.6510300289855071	0.6030956892145309
bc-s-dn-cf-ce-pme	0.5549617971014492	0.6505417101449275	0.6008542223796709
dn-bc-s-cf-pme-mi	0.5240054057971014	0.6851575797101448	0.5991880135574928
dn-bc-s-cf-emc-pme	0.5361403623188404	0.6687714347826085	0.5987949226177844
dn-bc-s-cf-pme-ce	0.5381846811594202	0.6648368985507246	0.5981680652371367
dn-bc-s-cf-pme-emc	0.5271564202898549	0.6784377826086957	0.598032468072919
bc-dn-s-cf-emc-pme	0.5371127826086955	0.6623270289855071	0.5964430513765383
dn-bc-s-cf-mi-pme	0.5255478405797103	0.6746048550724636	0.5954302014744647
bc-dn-s-cf-pme-mi	0.5240738405797102	0.6762302173913044	0.5953104796190919
bc-dn-s-cf-pme-emc	0.5269412318840581	0.6708279565217389	0.594547651405529
bc-dn-s-cf-pme-ce	0.5376781449275363	0.655668028985507	0.5937494164318998
bc-s-dn-cf-emc-pme	0.5338934637681159	0.6595714347826085	0.5934145919326493
dn-bc-s-lm-ce-pme	0.5657293913043477	0.6202206521739126	0.5923487587804445
bc-s-dn-cf-pme-mi	0.5203554347826088	0.6737713768115942	0.5921153584605571
bc-dn-s-cf-mi-pme	0.5246855652173911	0.6657308115942028	0.5910155219314775
bc-s-dn-cf-pme-emc	0.5211210724637682	0.6670515942028985	0.5895885363197632
bc-s-dn-cf-pme-ce	0.5330829710144928	0.6515787681159416	0.5893602850186034
bc-s-dn-cf-mi-pme	0.519592608695652	0.6629234347826086	0.5868987279285733
dn-bc-s-lm-pme-ce	0.5505265942028986	0.6233092463768113	0.5857886278710106
dn-bc-s-lm-emc-pme	0.5401064782608694	0.6350219999999996	0.5856445133681127
dn-bc-s-lm-pme-emc	0.5390458115942031	0.6358807246376809	0.585464637095554
dn-bc-s-lm-pme-mi	0.5327968985507248	0.6406862318840578	0.5842564824561012
bc-dn-s-lm-ce-pme	0.5613082463768118	0.608053101449275	0.5842133343894799
s-bc-dn-cf-ce-pme	0.4864046811594203	0.6965730434782608	0.5820793666823197
dn-bc-s-lm-mi-pme	0.5313953188405797	0.6349383913043475	0.5808642602977897
bc-s-dn-lm-ce-pme	0.556179811594203	0.6050154927536229	0.5800839618290475
s-dn-bc-cf-ce-pme	0.4842541014492754	0.6938614637681159	0.5796596067239013
dn-s-bc-cf-ce-pme	0.4833471304347826	0.6937004347826085	0.5790493196037233
s-bc-dn-cf-emc-pme	0.4665764347826086	0.7176447971014488	0.578650283739568

dn-s-bc-cf-emc-pme	0.4628727101449274	0.7153604347826082	0.5754309890666991
bc-dn-s-lm-emc-pme	0.5339958840579709	0.6195842028985503	0.5752003252564837
s-dn-bc-cf-emc-pme	0.46243682608695647	0.7147113043478256	0.5748989712558871
s-bc-dn-cf-mi-pme	0.45450026086956513	0.7261721594202893	0.5744958101611681
bc-dn-s-lm-mi-pme	0.5289267826086956	0.6230116231884057	0.574044887949422
bc-s-dn-lm-emc-pme	0.5301687681159419	0.6190058695652171	0.5728678550276045
s-bc-dn-lm-ce-pme	0.491521420289855	0.6662674492753622	0.5722628093461298
s-dn-bc-lm-ce-pme	0.4910954782608695	0.6659239999999998	0.5718673493611881
dn-s-bc-lm-ce-pme	0.49031399999999997	0.6662094492753621	0.5715346182970896
bc-dn-s-lm-pme-emc	0.5263500000000001	0.6200710144927535	0.5712918505267346
bc-dn-s-lm-pme-ce	0.538546927536232	0.6057705942028982	0.5711706332609972
s-dn-bc-cf-mi-pme	0.4506226521739129	0.7230661304347822	0.5708151867230321
bc-dn-s-lm-pme-mi	0.5219815942028988	0.6239103333333332	0.5706747851736532
bc-s-dn-lm-pme-ce	0.5354585507246378	0.605417144927536	0.5693643710373405
bc-s-dn-lm-pme-emc	0.5234567536231884	0.6189547826086954	0.5692065188873842
bc-s-dn-lm-mi-pme	0.5224840869565218	0.6195657246376811	0.5689580230094964
dn-s-bc-cf-mi-pme	0.4490036956521738	0.7208721884057967	0.5689237881184727
bc-s-dn-lm-pme-mi	0.5191962318840581	0.6227941159420288	0.5686407989554254
s-bc-dn-lm-emc-pme	0.4680739710144927	0.6875262028985505	0.5672857480734382
dn-s-bc-lm-emc-pme	0.4667260724637681	0.686273289855072	0.5659519742971544
s-dn-bc-lm-emc-pme	0.4651642898550726	0.6836947536231881	0.5639418272718997
s-bc-dn-lm-mi-pme	0.4587003333333333	0.6905386811594201	0.5628057597674231
dn-s-bc-lm-mi-pme	0.4566958405797102	0.6885229999999996	0.5607544830346554
s-dn-bc-lm-mi-pme	0.4557548260869566	0.6867072318840577	0.559437337902981
s-dn-bc-cf-pme-ce	0.4372121884057971	0.713868797101449	0.5586699732537435
s-dn-bc-cf-pme-emc	0.42720565217391293	0.7301428115942026	0.558499002691317
s-dn-bc-cf-pme-mi	0.42341304347826086	0.7337314782608694	0.5573791154198712
s-bc-dn-cf-pme-emc	0.42746657971014485	0.7260800869565217	0.5571130687454288
s-bc-dn-cf-pme-ce	0.4367492898550725	0.7106041304347824	0.5570959067660366
s-bc-dn-cf-pme-mi	0.42346782608695643	0.7305745507246377	0.5562147218384437
dn-s-bc-cf-pme-emc	0.4252076231884057	0.7274809130434778	0.5561748195937573
dn-s-bc-cf-pme-mi	0.4211458550724637	0.7310695797101446	0.5548755926011664
dn-s-bc-cf-pme-ce	0.4332279710144927	0.7102462898550721	0.5547058311163532
s-bc-dn-lm-pme-emc	0.43603172463768103	0.699469144927536	0.5522596650069581
s-dn-bc-lm-pme-emc	0.43564389855072455	0.6995223478260868	0.5520350013634212
dn-s-bc-lm-pme-emc	0.43495657971014484	0.6998402753623186	0.5517246890569607
dn-s-bc-lm-pme-mi	0.4306661449275362	0.7014965797101447	0.5496460931032078
s-dn-bc-lm-pme-mi	0.4314394057971013	0.7002124782608694	0.5496355661277618
s-bc-dn-lm-pme-mi	0.43180940579710136	0.699469144927536	0.5495792534699303
dn-s-bc-lm-pme-ce	0.4421017536231883	0.6799505797101447	0.5482767035603086
s-dn-bc-lm-pme-ce	0.4419092028985506	0.6778139710144926	0.5472953787896584
s-bc-dn-lm-pme-ce	0.44089517391304334	0.6771568985507246	0.5464020576031342

Supplement C:
This table shows the results of comparing all possible pipeline instances for an intensity threshold I = 15. The instances are sorted by their geometric mean. The highlighting indicates the division into the two groups. The rows with blue characters contain the pareto-optimal instances.

Pipeline instance	Average PPV	Average SENS	Geometric Mean
dn-s-bc-cf-ce-e	0.8245790579710142	0.6259132318840578	0.718411402414069
s-dn-bc-cf-ce-e	0.8252472608695649	0.6246192463768115	0.7179591368308336
s-bc-dn-cf-ce-e	0.8252472608695649	0.6246192463768115	0.7179591368308336
s-dn-bc-cf-emc-e	0.8134643478260867	0.6336351739130431	0.7179412395919618
s-bc-dn-cf-emc-e	0.8134643478260867	0.6336351739130431	0.7179412395919618
dn-s-bc-cf-emc-e	0.80910984057971	0.6342390434782607	0.7163581864948032
s-dn-bc-cf-mi-e	0.7883070579710142	0.6342652898550721	0.7071038145978173
s-bc-dn-cf-mi-e	0.7883070579710142	0.6342652898550721	0.7071038145978173
dn-s-bc-cf-mi-e	0.7849295942028981	0.6348691594202897	0.7059232193912472
dn-bc-s-cf-emc-e	0.8528249710144924	0.5688647681159419	0.6965214134394176
dn-bc-s-cf-ce-e	0.8617650724637678	0.5614259710144929	0.695569761127053
bc-dn-s-cf-emc-e	0.8530536376811589	0.5665566666666666	0.6952001333806774
bc-s-dn-cf-emc-e	0.848433942028985	0.5665566666666666	0.6933151563919554
bc-dn-s-cf-ce-e	0.8619561449275359	0.5575752173913043	0.6932570842694227
dn-s-bc-lm-ce-e	0.8242948550724637	0.5826734492753622	0.6930329908633551
s-dn-bc-lm-ce-e	0.8245437101449276	0.5819833188405797	0.6927269916491416
s-bc-dn-lm-ce-e	0.824342420289855	0.5814465507246377	0.6923229426312086
bc-s-dn-cf-ce-e	0.8583490724637677	0.5582653478260869	0.6922330124280295
dn-s-bc-lm-emc-e	0.8013756086956519	0.5896707536231882	0.6874210930098132
s-dn-bc-lm-emc-e	0.8003124782608694	0.5889806231884057	0.6865628464980786
s-bc-dn-lm-emc-e	0.800111188405797	0.5884438550724637	0.686163619111446
dn-bc-s-cf-mi-e	0.8247299710144925	0.5697172898550724	0.6854654797643928
s-dn-bc-lm-mi-e	0.7961349710144926	0.5896707536231882	0.6851696931008339
s-bc-dn-lm-mi-e	0.7959336811594202	0.5891339855072463	0.6847711893624783
dn-s-bc-lm-mi-e	0.795080956521739	0.5896707536231882	0.6847159898991838
bc-dn-s-cf-mi-e	0.8245124782608693	0.5665566666666666	0.6834713171074193
bc-s-dn-cf-mi-e	0.8199963043478258	0.5665566666666666	0.6815969284483973
dn-bc-s-lm-ce-e	0.8557945797101447	0.5301775797101449	0.6735897111742319
bc-dn-s-lm-ce-e	0.8552722753623186	0.5286746231884057	0.6724289909727749
bc-s-dn-lm-ce-e	0.8538229999999998	0.5286746231884057	0.6718590274712353
dn-bc-s-lm-emc-e	0.8325144782608693	0.5373474492753624	0.6688419330292377
bc-dn-s-lm-emc-e	0.8311138260869562	0.5358444927536232	0.6673438143566538
bc-s-dn-lm-emc-e	0.8305428985507244	0.5358444927536232	0.6671145615140153
dn-bc-s-lm-mi-e	0.8237183913043474	0.5373474492753624	0.66529916315038
bc-dn-s-lm-mi-e	0.8223177391304345	0.5358444927536232	0.6638030067773526
bc-s-dn-lm-mi-e	0.8217468115942026	0.5358444927536232	0.6635725306480089
dn-bc-s-cf-ce-pme	0.5784792898550727	0.4999642463768115	0.5377908163933115
dn-s-bc-cf-ce-pme	0.5314083913043479	0.5439207971014491	0.5376281947448996
dn-bc-s-cf-pme-ce	0.5780370000000001	0.49945966666666675	0.5373138443600725
s-dn-bc-cf-ce-pme	0.5325641739130436	0.5416116666666666	0.537068868805482
dn-bc-s-cf-pme-emc	0.5691842463768119	0.5041702028985506	0.5356918302368031
dn-bc-s-lm-ce-pme	0.6025968695652175	0.47584734782608706	0.5354849411429898
bc-s-dn-cf-pme-ce	0.5727469855072466	0.4994185217391306	0.5348274982015906
bc-s-dn-cf-ce-pme	0.5735858695652175	0.4973795507246377	0.534125343066914
bc-dn-s-cf-pme-ce	0.5728688260869567	0.49678376811594216	0.5334715869281462
dn-s-bc-cf-emc-pme	0.5114365362318841	0.5550702463768116	0.5328069107775677
bc-dn-s-cf-ce-pme	0.5731716086956521	0.4951148550724638	0.5327154756255901
dn-bc-s-cf-pme-mi	0.5613866086956525	0.5050227246376812	0.5324593831444668
bc-s-dn-cf-pme-emc	0.5598327246376813	0.5051642463768117	0.5317964614762601
bc-dn-s-lm-ce-pme	0.5973862028985509	0.4725346811594203	0.5313056548877525
bc-s-dn-cf-pme-mi	0.5565426231884059	0.5060167681159421	0.5306787158955649
s-bc-dn-cf-ce-pme	0.5250392753623189	0.5360374492753622	0.530509862240658
dn-bc-s-lm-pme-emc	0.5884237536231884	0.4776976811594203	0.5301779537522464
bc-dn-s-cf-pme-emc	0.55921715942029	0.5025295072463769	0.5301161415833298
s-bc-dn-cf-emc-pme	0.509726	0.5510571449275362	0.5299888246513631
dn-bc-s-lm-pme-ce	0.594711231884058	0.47164313043478273	0.5296144513799855
bc-dn-s-cf-pme-mi	0.5557832898550725	0.5033820289855073	0.5289341359030316
dn-bc-s-cf-emc-pme	0.5520648550724638	0.5055104782608696	0.5282750883003086
dn-s-bc-cf-pme-ce	0.5110069130434783	0.545554507246377	0.5279982240925882
dn-s-bc-cf-mi-pme	0.5029203188405796	0.5541245072463769	0.5279019547811193

s-dn-bc-cf-emc-pme	0.5082647246376812	0.5478179420289855	0.5276708590181417
dn-bc-s-lm-pme-mi	0.5802942608695653	0.47975794202898553	0.527636977822834
s-bc-dn-cf-pme-emc	0.5054517101449274	0.5503777246376812	0.5274365953778615
dn-bc-s-lm-emc-pme	0.5762664782608697	0.48268082608695656	0.5274019148364725
bc-s-dn-lm-ce-pme	0.5930593188405798	0.4688452028985508	0.5273073265873404
bc-s-dn-cf-emc-pme	0.5501680289855074	0.5053020144927537	0.5272580140271788
s-bc-dn-cf-pme-ce	0.5108654347826086	0.5433935652173913	0.5268785343443538
bc-s-dn-cf-mi-pme	0.5470430000000001	0.5070108115942029	0.526646670365367
dn-bc-s-cf-mi-pme	0.5476085507246377	0.5063978695652174	0.5266002311456507
dn-s-bc-cf-pme-emc	0.5037324492753623	0.5502879855072466	0.5264958829339222
s-bc-dn-cf-mi-pme	0.5024391014492754	0.5510865362318841	0.5262009350857725
s-dn-bc-cf-pme-ce	0.5101143333333333	0.5420853043478262	0.5258569041452137
s-bc-dn-cf-pme-mi	0.501101884057971	0.5503777246376812	0.5251621794831395
dn-s-bc-cf-pme-mi	0.5010776666666666	0.5502879855072466	0.5251066746601795
bc-dn-s-cf-mi-pme	0.5447504782608696	0.5047786376811595	0.5243838329817977
bc-dn-s-cf-emc-pme	0.5470240579710145	0.5023659565217393	0.5242196716291865
s-dn-bc-cf-pme-emc	0.5026362028985507	0.5459129855072464	0.5238278630889864
s-dn-bc-cf-mi-pme	0.4997705652173913	0.5474269565217391	0.5230562871012123
dn-s-bc-lm-emc-pme	0.525939536231884	0.519443507246377	0.5226814300315469
dn-s-bc-lm-ce-pme	0.5410052173913044	0.5048381594202899	0.5226089151407579
s-dn-bc-cf-pme-mi	0.4992442753623188	0.5459129855072464	0.5220574037981315
dn-bc-s-lm-mi-pme	0.5670590144927538	0.4799238840579711	0.521675344180121
s-dn-bc-lm-ce-pme	0.539716956521739	0.5019576811594202	0.5204950258917675
dn-s-bc-lm-pme-ce	0.5261620724637682	0.5134417971014494	0.5197630229752981
bc-s-dn-lm-pme-emc	0.573800304347826	0.4699802753623189	0.5193022482527624
s-dn-bc-lm-emc-pme	0.5221724492753623	0.5163422753623189	0.5192491796722992
bc-dn-s-lm-pme-emc	0.5737808115942027	0.46901408695652175	0.5187593695183027
dn-s-bc-lm-pme-emc	0.5206675797101449	0.5165306521739133	0.5185949908295433
bc-s-dn-lm-pme-ce	0.5811313623188404	0.46244559420289866	0.5184029688933852
bc-s-dn-lm-pme-mi	0.5689384057971013	0.47083279710144926	0.5175662865565034
dn-s-bc-lm-mi-pme	0.5180474927536232	0.516630695652174	0.5173386091934072
bc-dn-s-lm-pme-ce	0.5800766666666666	0.46071662318840595	0.5169632124794309
dn-s-bc-lm-pme-mi	0.517370492753623	0.5165306521739133	0.5169504019125701
bc-dn-s-lm-pme-mi	0.5684358260869564	0.4698666086956522	0.5168065536200076
s-dn-bc-lm-pme-ce	0.5234704057971015	0.5095777826086958	0.5164773844490784
s-bc-dn-lm-emc-pme	0.5170164492753622	0.5137831594202901	0.5153972688916344
bc-s-dn-lm-emc-pme	0.5628935072463769	0.4717480144927536	0.5153095132193279
s-bc-dn-lm-pme-emc	0.5150295942028983	0.5153256666666668	0.5151776091656851
s-dn-bc-lm-pme-emc	0.5176611304347825	0.5126666376811595	0.5151578313470815
s-bc-dn-lm-pme-ce	0.519726536231884	0.5100867681159422	0.514884092947738
s-bc-dn-lm-ce-pme	0.5320244057971014	0.4976286086956522	0.5145391771759878
s-bc-dn-lm-pme-mi	0.5120373478260867	0.5153256666666668	0.5136788759786702
s-dn-bc-lm-pme-mi	0.5142542463768115	0.5126666376811595	0.513459828422106
s-dn-bc-lm-mi-pme	0.5141900579710146	0.5122119275362319	0.5132000396661133
bc-dn-s-lm-emc-pme	0.5595773623188406	0.4692248115942029	0.5124134877288448
bc-s-dn-lm-mi-pme	0.5557555942028987	0.47216685507246375	0.51225908591621
s-bc-dn-lm-mi-pme	0.5108768985507246	0.5120055507246378	0.5114409132978693
bc-dn-s-lm-mi-pme	0.5543717681159421	0.47034815942028996	0.5106346450916792

Supplement D:
The Figure shows the peak candidate detection step and the peak picking of the best pipeline instance.

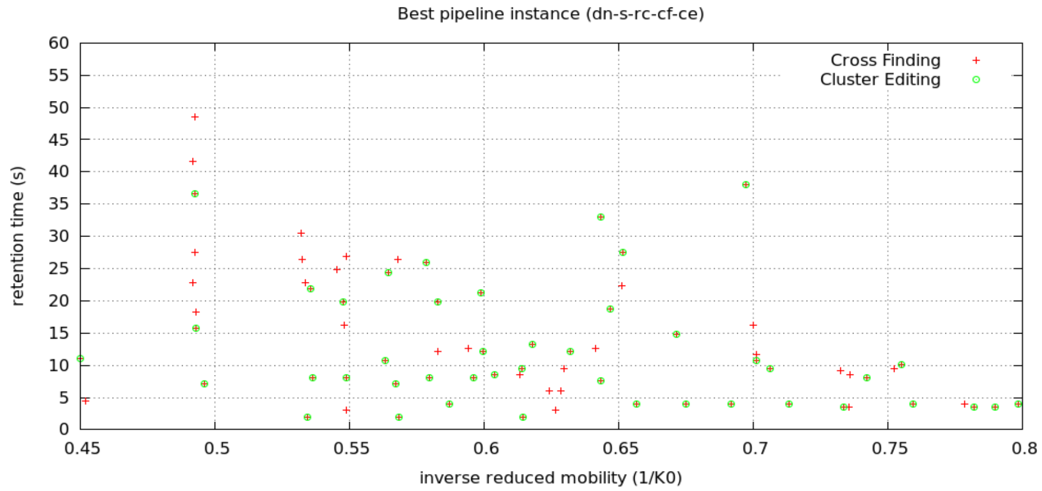

Supplement E:
Histogram of an entire measurement for the De-Noising module. By estimating the probability that a data point belongs to the background noise (which is visible in the histogram at signal intensity s = 0) with the help of EM algorithm, data points are multiplied with their counter probability, to eliminate the influence background noise.

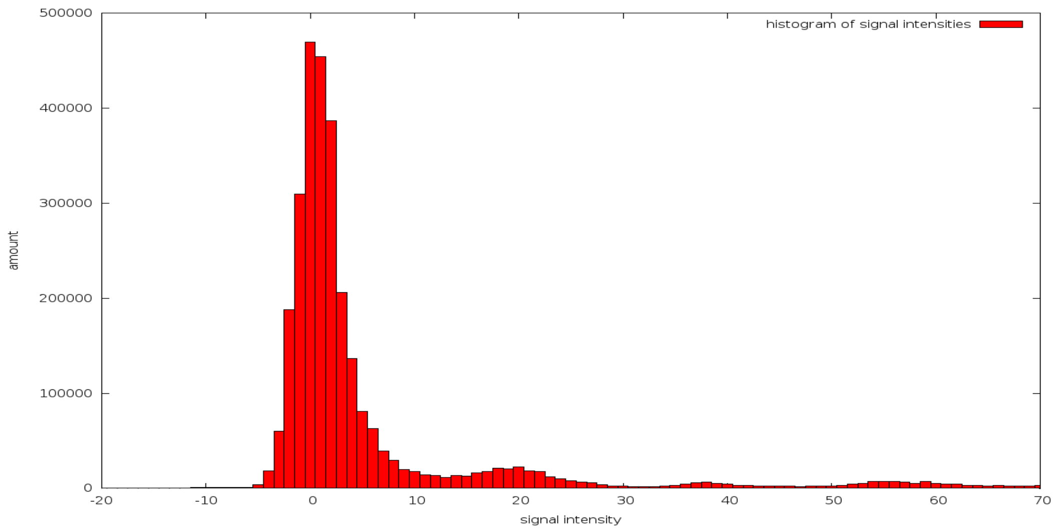

Supplement: Additional file 1 — Supplement. [file 1471-2105-15-25-S1.pdf]
